# AI-Based Assessment Tools in Pharmacy Education

**DOI:** 10.21203/rs.3.rs-10463681/v1

**Published:** 2026-08-03

**Authors:** Keykavous Parang

**Affiliations:** Chapman University School of Pharmacy, Harry and Diane Rinker Health Science Campus

**Keywords:** artificial intelligence, adaptive learning, assessment, Doctor of Pharmacy, objective structured clinical examination

## Abstract

**Purpose:**

Integrating artificial intelligence (AI) into pharmacy education represents a shift in assessment practice, offering opportunities to enhance efficiency, objectivity, and personalization in evaluating student competencies. As Doctor of Pharmacy (PharmD) programs in North America increasingly adopt AI-powered tools, understanding their capabilities, effectiveness, and limitations has become critical for educators and administrators. AI-based assessment tools in pharmacy education were examined by synthesizing their technical features, pedagogical effectiveness, implementation challenges, ethical considerations, and future directions.

**Method:**

A focused literature search was conducted in Google Scholar and PubMed using keywords including AI-based assessment, artificial intelligence, pharmacy education, automated grading, ChatGPT, GPT-4, machine learning, adaptive learning, computer-based testing, and virtual OSCE. From 600 initial results, 222 unique records remained after deduplication and were ranked by relevance; the 30 most relevant articles published between 2004 and 2026 were selected for detailed synthesis using a structured data-extraction approach.

**Results:**

Four categories of AI assessment tools emerged: generative AI tools for grading and feedback; automated assessment systems for objective scoring; adaptive learning platforms for personalized progression; and virtual assessment environments, including virtual objective structured clinical examinations (OSCEs). GPT-4 showed substantial agreement with human raters, and automated short-answer grading correlated highly with human examiners. Recurring challenges included algorithmic transparency, data privacy, academic-integrity risks, validation requirements, and faculty-training needs.

**Conclusion:**

AI-based assessment tools show promise in pharmacy education, offering greater efficiency and objectivity while maintaining acceptable accuracy across assessment types. Successful implementation requires attention to validation, ethics, faculty development, and integration with existing curricula and accreditation frameworks. Future work should prioritize longitudinal effectiveness, bias mitigation, and pharmacy-specific AI assessment frameworks.

## Teaser Text

Artificial intelligence has moved quickly from novelty to a working tool in the assessment of health professions students. Drawing on 30 studies from pharmacy education, this review maps where AI already scores structured tasks almost as reliably as human examiners, where it still falls short on judgment and nuance, and what programs must put in place before trusting it, neither hype nor dismissal, but a practical account of what these systems can do today.

## Introduction

### Current Status of Assessment in Pharmacy Education

Pharmacy education has entered what has been described as a “golden age of assessment,” marked by a shift from knowledge-based testing toward competency-based evaluation.^1^ Contemporary PharmD curricula in North America must assess not only foundational knowledge but also clinical reasoning, professional judgment, communication, and adaptive problem-solving. Accreditation Council for Pharmacy Education (ACPE) standards require assessment across the cognitive, affective, and psychomotor domains, prompting educators to employ diverse methods, such as objective structured clinical examinations (OSCEs), performance-based assessments, reflective writing, and clinical competency evaluations.^2^

These methods have grown more prevalent. Over 80% of pharmacy programs now use OSCEs^3^, but these assessments demand substantial faculty time, and written assessments such as short-answer and case-based items require extensive manual grading, particularly in large cohorts. The volume and complexity of assessments create workload challenges while demanding consistency, objectivity, and timely feedback.^4^ Computer-based testing is now standard in many programs; as early as 2006, the University of Manchester implemented online summative assessment with automated scoring and immediate reporting.^5^ The COVID-19 pandemic accelerated the adoption of digital tools, including virtual OSCEs and remote proctoring, building infrastructure that now facilitates deeper AI integration.^6^ The release of ChatGPT in November 2022 and GPT-4 in 2023 then catalyzed urgent discussion about academic integrity and appropriate AI use.

### Major Challenges in Traditional Assessment

Traditional assessment faces several interconnected challenges. Manual grading of written responses is time-consuming and prone to inter-rater variability driven by fatigue, implicit bias, and subjective rubric interpretation.^4,7^ Timely, individualized feedback is difficult to provide at scale, limiting its educational value, even though feedback is most effective when prompt and tailored.^8^ One-size-fits-all methods struggle to accommodate diverse learning paces,^9^ performance-based assessments such as OSCEs are resource-intensive, requiring standardized patients, trained evaluators, and dedicated facilities,^10^ and maintaining academic integrity has grown more complex in digital environments where online resources and AI tools create new opportunities for misconduct.^11^

## Emergence of AI in Educational Assessment

Large language models (LLMs) such as ChatGPT and GPT-4 demonstrate strong natural-language understanding, generation, and reasoning, sparking interest in educational applications.^12^ In pharmacy education, AI offers potential solutions through automated grading, personalized feedback, adaptive testing that adjusts difficulty to performance, item creation, and analysis of complex student work.^13^ Although computer-based testing and automated scoring are decades old, advances in machine learning and natural-language processing now enable more sophisticated evaluation of open-ended responses, clinical reasoning, and contextual feedback.^14^

## Objectives

This narrative review synthesizes the current evidence on AI-based assessment tools in pharmacy education. Its objectives are to (1) characterize the major categories of AI assessment tools and their functions; (2) evaluate their effectiveness, validity, and reliability across assessment types; (3) examine implementation, ethical, and academic-integrity considerations; and (4) identify research gaps and propose future research, technology, and policy directions relevant to PharmD programs and accreditation.

## Method

### Search Strategy

Because this is an unstructured (narrative) review, articles were selected to provide a focused synthesis rather than through an exhaustive systematic protocol. Google Scholar and PubMed were searched between January and March 2026, with no lower date restriction, to capture the historical development of AI assessment tools in pharmacy education. The following keywords and search terms were used in various combinations:

1. Core terms: AI-based assessment, artificial intelligence, pharmacy education, pharmacy students; 2. Technology-specific terms: automated grading, machine learning, natural language processing, ChatGPT, GPT-4, large language models, generative AI; 3. Assessment-specific terms: adaptive learning, computer-based testing, automated evaluation, virtual OSCE, objective structured clinical examination, competency-based assessment; and 4. Educational context terms: PharmD, pharmaceutical education, pharmacy curriculum, pharmacy training

Boolean operators (AND, OR) were used to strategically combine search terms. Example queries include: “(AI OR artificial intelligence OR machine learning) AND (assessment OR evaluation OR grading) AND (pharmacy education OR pharmacy students)” and “(ChatGPT OR GPT-4 OR generative AI) AND (automated grading OR assessment) AND pharmacy.”

### Study Selection and Inclusion/Exclusion Criteria

The initial search yielded approximately 600 results; deduplication produced 222 unique records, which were ranked using multi-criteria relevance (topical relevance, recency, citation metrics, journal quality, and methodological rigor). Articles were included if they addressed AI, machine learning, or automated systems applied to educational assessment; addressed or were clearly applicable to pharmacy education; provided empirical data, systematic analysis, or substantive frameworks; were peer-reviewed or reputable preprints; and were in English. Articles were excluded if they addressed only clinical practice without an assessment component, general education without pharmacy relevance, opinion pieces without analysis, or lacked methodological detail. The 30 most relevant articles were selected for detailed synthesis, representing diverse designs, technologies, assessment contexts, and settings.

## Data Extraction and Analysis

For each article, a standardized protocol captured 1. bibliographic information; 2. Study design and methodology: research design type (e.g., pilot study, cross-sectional, experimental, qualitative), sample size, setting, data collection methods; 3. AI tools and technologies: specific AI systems evaluated, types of AI technologies employed, primary functions in assessment; 4. Key findings and outcomes: quantitative results (accuracy rates, correlation coefficients, agreement statistics), qualitative findings, and effectiveness measures; and 5. Limitations and challenges: methodological limitations, technical challenges, ethical concerns, and implementation barriers

Thematic analysis identified patterns across the literature; quantitative findings (accuracy rates, correlations, and agreement statistics) were synthesized narratively, and qualitative findings on implementation and user perceptions were analyzed using constant comparison. The framework was guided by established criteria for evaluating assessment tools: validity, reliability, fairness, feasibility, and educational impact.

## Results

### Overview of Reviewed Literature

**Figure 1:**
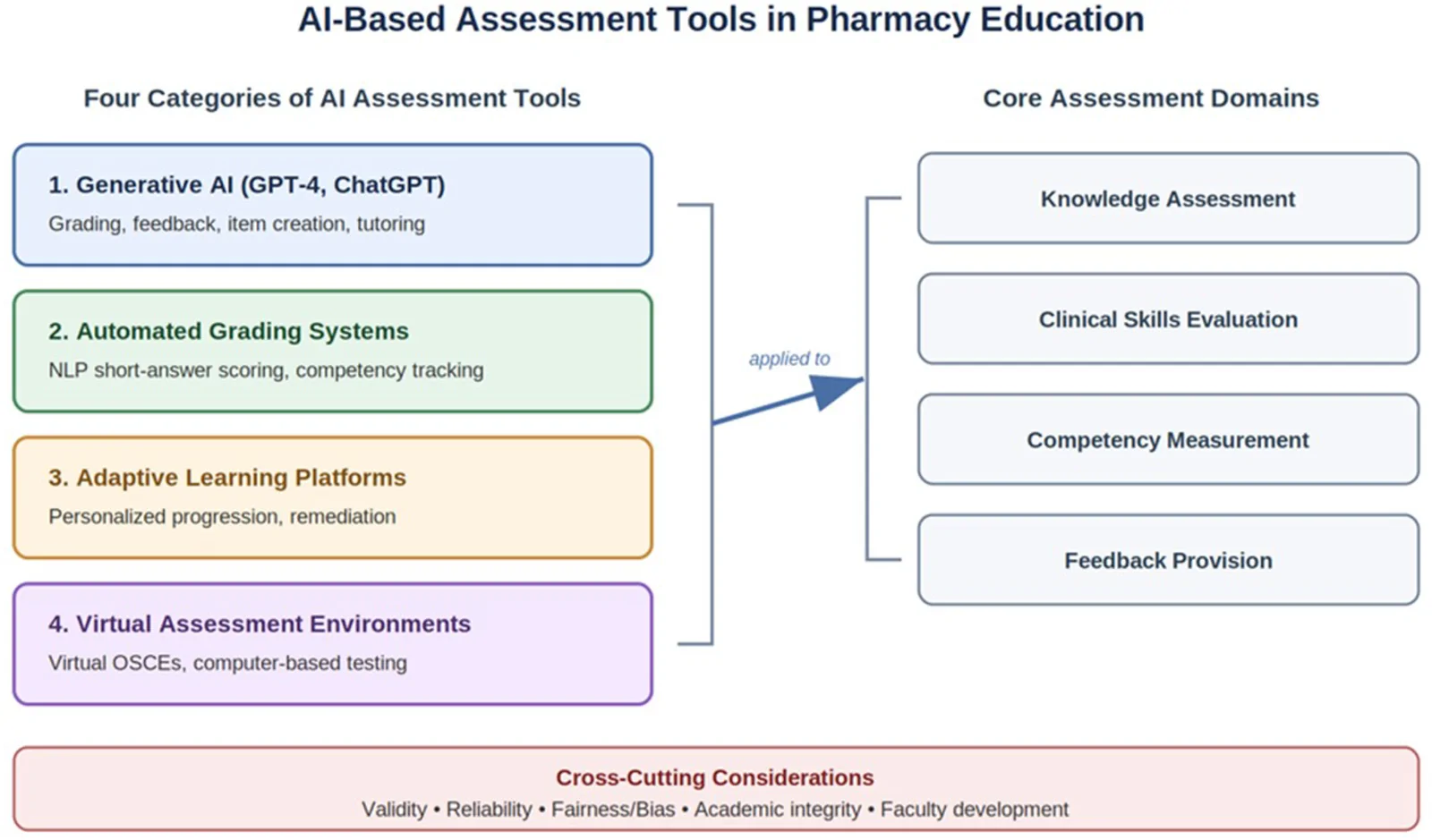
Conceptual framework of AI-based assessment tools in pharmacy education. Four categories of AI assessment tools (generative AI, automated grading systems, adaptive learning platforms, and virtual assessment environments) are applied across four core assessment domains (knowledge assessment, clinical skills evaluation, competency measurement, and feedback provision). Cross-cutting considerations (validity, reliability, fairness and bias, academic integrity, and faculty development) apply across all categories.

The 30 reviewed articles were published between 2004 and 2026, with a marked acceleration in 2023–2024 coinciding with the emergence of ChatGPT and GPT-4. Designs included pilot, cross-sectional, randomized, qualitative, systematic review, and implementation studies. Four categories of AI-based assessment tools were identified: generative AI tools for grading, feedback, item creation, and tutoring (n = 12); automated assessment systems, including natural-language-processing short-answer graders and competency-tracking systems (n = 7); adaptive learning platforms using item response theory and reinforcement learning (n = 4); and virtual assessment environments, including virtual OSCEs and computer-based testing (n = 7). Figure 1 presents a conceptual framework illustrating these categories and their relationships to core assessment domains in pharmacy education. Table 1 provides a comparative overview of the six AI tool categories, covering their specific tools, primary functions, key advantages, and principal limitations, spanning a spectrum from established computer-based testing (CBT) platforms to emerging generative AI.

### Generative AI Tools for Grading and Feedback

Table 2 exhibits a detailed study-level summary of all 30 reviewed papers, capturing study design, AI tool used, sample/setting, key findings, and limitations, to allow readers to compare across the full breadth of the literature. We have highlighted some of these tools below:

### Grading Performance and Accuracy

Multiple studies evaluated GPT-4 for grading pharmacy examinations. In a cross-sectional study, GPT-4 showed substantial agreement with human raters (intraclass correlation coefficient 0.62–0.93) across item types, and optimized prompts improved consistency; occasional out-of-range scores and misinterpretation of nuanced reasoning decreased with refined prompting.^4^ A pilot study grading pharmacy OSCEs with LLMs reported 94%–96% accuracy versus human evaluators while reducing grading time, with particular strength in applying consistent rubric criteria; human oversight remained essential for complex scenarios.^15^ Evaluation of LLM performance on critical-care assessments found high reproducibility but accuracy varying by question complexity.^16^ Comparative studies revealed generational differences: on the Taiwan National Pharmacist Licensing Examination, GPT-4 achieved passing scores whereas GPT-3.5 did not;^17^ across four AI tools on pharmacology multiple-choice questions, accuracy ranged from 69% to 88%;^18^ and a comparison of ChatGPT and Gemini across Bloom’s taxonomy found stronger performance on lower-order recall than higher-order reasoning.^35^

### Feedback Generation, Educational Support, and Exam Item Generation

Beyond grading, generative AI provided personalized feedback. A randomized study found that AI-assisted peer feedback was more comprehensive and specific than self-generated peer feedback, though students questioned its authenticity; a hybrid AI-plus-human approach was recommended.^19^ A proof-of-concept study showed ChatGPT could provide effective concept explanation and 24/7 tutoring,^20^ an AI chatbot for written-communication training in self-care was well received as a supplementary resource,^21^ and ChatGPT-driven active recall was feasible when students developed prompting proficiency.^28^ For item creation, ChatGPT produced grammatically correct multiple-choice questions rapidly but required expert review for distractor quality;^22^ AI-generated pharmacy examination questions were generally content-accurate but lacked clinical nuance, best used as a starting point for faculty refinement;^13^ and an exploratory assessment of GPT-4 for valid exam items emphasized the need for psychometric evaluation and oversight.^36^

### Automated Grading and Short-Answer Systems

An AI-assisted automated short-answer scoring tool correlated highly with human examiners (correlation coefficient > 0.93), performing best for questions with clearly defined answers and structured formats and less well for creative or multi-approach items.^7^ A comparison of semantic-similarity algorithms found word-embedding methods (Word2Vec, GloVe) outperformed keyword matching, though pharmacy-specific terminology challenged general-purpose models.^23^ Machine-learning classification of pharmacy students’ reflective statements achieved moderate accuracy (ensemble F-score ≈ 0.79), identifying linguistic features predictive of reflection quality but remaining below expert classification.^14^ An early rule-based competency assessment program for Advanced Pharmacy Practice Experiences reduced administrative burden and improved documentation consistency, though effectiveness depended on preceptor training.^24^

### Adaptive Learning Platforms

An adaptive learning platform used with first-year pharmacy students identified knowledge gaps, adjusted difficulty levels, and provided personalized remediation, resulting in improved examination performance and generally positive student feedback on self-paced learning; some students reported frustration with algorithmic opacity.^9^ Such platforms can accommodate diverse backgrounds and paces, but implementation challenges include algorithmic bias and equitable access.

### Virtual Assessment Environments

A virtual OSCE for pharmacy students achieved validity comparable to in-person OSCEs while improving scalability, scheduling, and geographic access; students were satisfied, though the lack of physical presence altered interaction dynamics, and video recording aided consistent review despite time-intensive grading.^6^ A qualitative study of AI integration into pharmacy OSCEs found cautiously optimistic perspectives, with stakeholders emphasizing that AI should augment rather than replace human evaluation, particularly for interpersonal skills and ethical judgment.^25^ Digitizing OSCE evaluation with electronic scoring software reduced transcription errors, enabled real-time tracking, and supported psychometric analysis, though it did not employ advanced AI.^26^ For computer-based testing, eliminating backward navigation reduced completion time without affecting scores;^27^ early online summative assessment enabled immediate reporting that students valued;^5^ virtual interactive cases enhanced clinical-reasoning practice with positive student experience;^33^ and student perceptions of AI-supported formative assessment were generally positive but highlighted trust and transparency concerns.^29^

## Discussion

### Effectiveness and Validity

The synthesized evidence indicates that AI-based assessment tools have reached a substantial level of maturity across several applications. Generative AI, particularly GPT-4, demonstrates grading agreement approaching human performance for many tasks (intraclass correlation coefficient 0.62–0.93),^4^ and automated short-answer grading shows strong correlation with human scoring (> 0.93),^7^ suggesting AI tools have crossed a threshold of technical capability that makes practical implementation viable. Effectiveness nonetheless varies by assessment type, content domain, and complexity: accuracy is highest for knowledge-based questions with defined answers and structured formats, and decreases for tasks requiring professional judgment, creative problem-solving, or evaluation of nuanced behaviors.^16^ This pattern supports a complementary model in which AI handles routine, objective scoring while human evaluators focus on judgment-intensive assessment.

Validity requires consideration beyond accuracy. Content, construct, and criterion validity remain applicable, but AI introduces additional concerns: a tool may show high criterion validity yet lack content validity if its training data underrepresent the competency domain, and the “black box” nature of many algorithms raises questions about construct validity, namely whether the intended construct is being measured.^35^ These considerations underscore the need for rigorous validation specific to each tool, context, and intended use.

### Implementation Considerations

Successful implementation requires attention across technical, pedagogical, and organizational dimensions. Faculty development is a prerequisite: inadequate training leads to suboptimal use and resistance, and faculty must understand AI capabilities and limitations, craft effective prompts, interpret results critically, and integrate tools within broader assessment strategies.^25^ Reliable computing infrastructure, data security, technical support, and integration with learning-management systems are essential, and institutions must invest in both implementation and ongoing maintenance.^37^ Validation and quality assurance should precede deployment, with ongoing monitoring and audits to detect bias and errors; the FLUF (Flawed, Limited, Uninformative, or Fabricated) framework offers a structured approach to evaluating AI-generated outputs in pharmacy contexts.^30^ Students need clear communication about how tools are used and support for critical-evaluation skills, as student trust affects engagement and outcomes.^31^

### Ethical and Academic-Integrity Considerations

Integrating AI raises significant ethical questions. Academic-integrity concerns are paramount, because tools that can grade assessments can also complete them; programs must update policies to address AI use, consider detection mechanisms, design AI-resistant assessments, and educate students on appropriate use.^12,38^ Algorithmic bias is a critical concern: systems trained on historical data may disadvantage students by linguistic, cultural, or demographic factors unrelated to competency, making bias testing across diverse populations essential.^11^ Transparency and explainability pose additional challenges, as opaque models make it difficult for students to understand scores, for faculty to explain decisions, and for institutions to verify that appropriate criteria are met; more interpretable models and clear documentation are priorities.^11^ Data-privacy obligations require compliance with regulations such as FERPA and GDPR, robust security, informed consent, and clear retention policies. Stakeholders must also decide which functions should remain human, how to preserve meaningful oversight,^36^ and how to ensure efficiency gains do not erode educational quality or humanistic values.

### Research Gaps and Limitations of the Evidence Base

The evidence base, though growing, has notable limitations. Many studies use pilot or proof-of-concept designs with small samples and short time frames, limiting generalizability. Publication bias likely favors positive findings, and many studies lack appropriate comparison groups,^32^ making it difficult to isolate the effect of AI tools. Outcome measures, such as technical performance, satisfaction, or learning outcomes, vary widely, hindering synthesis, and most studies originate from single institutions or from tool developers. Key knowledge gaps remain (Table 3): long-term effects on learning are largely unknown; optimal human–AI integration strategies are unclear; comparative effectiveness of different tools is understudied; differential impacts across student subgroups are rarely examined despite pressing equity concerns;^11^ faculty development needs are underexplored; the influence of AI on student learning behaviors is largely unknown; and formal cost-effectiveness analyses are absent.^39^

Bias and equity warrant particular attention. AI can perpetuate bias through unrepresentative training data, design choices that favor particular response styles, evaluation criteria that privilege certain backgrounds, and unequal access to technology.^11^ In pharmacy education specifically, potential sources include linguistic bias affecting non-native English speakers, cultural bias in communication expectations, and disability-related bias when accessibility is not designed in. Transparency about training and validation data, diverse development teams, and ongoing monitoring for disparate impact should be standard practice.

### Future Directions

Several priorities emerge (Table 4). Longitudinal studies should track students through AI-enhanced programs into practice, examining learning outcomes, competency development, and professional identity with rigorous comparison groups. Bias and equity research should identify sources of bias and test mitigation strategies, and comparative-effectiveness studies should evaluate different tools on identical tasks. Technology priorities include pharmacy-specific models trained on curated education data and calibrated to ACPE competency frameworks; explainable AI that provides interpretable rationale for assessment decisions; and multimodal systems integrating video, audio, and text to assess clinical and communication skills, particularly for AI-enhanced OSCEs. Advanced adaptive systems using deep reinforcement learning could personalize assessment in real time, and robust integrity tools paired with assessment redesign could reduce AI-assisted misconduct.

At the institutional level, programs should adopt a phased approach, beginning with low-stakes formative applications, invest in faculty training, establish clear, acceptable-use policies, and maintain ongoing quality assurance; AI should augment rather than replace human judgment in high-stakes decisions. At the national level, organizations, including ACPE, should develop standardized guidance on validation, ethical use, and accreditation compliance; facilitate resource sharing; and support the integration of AI-literacy competencies into PharmD curricula so graduates are prepared for AI-enabled practice.

### Implications for wider health professions education

Although the review is anchored in pharmacy education, the tool categories, implementation barriers, and research priorities it identifies are directly transferable to medical, nursing, dental, and other health professions programs that rely on competency-based and performance-based assessment, including objective structured clinical examinations. The tasks that AI currently performs well, such as scoring structured knowledge items and applying explicit rubric criteria, and those it performs poorly, such as judging professional reasoning, communication, and interpersonal behavior, are defined by the nature of the assessment task rather than by disciplinary content. The same applies to the cross-cutting constraints of validity, bias, transparency, and faculty readiness. Evidence generated in pharmacy education can therefore inform adoption decisions across the health professions, and evidence generated in medical, nursing, and dental education is likely to be equally informative for pharmacy. Cross-professional collaboration on validation standards and shared evaluation frameworks would accelerate progress across these disciplines and reduce duplicated effort.

## Conclusion

This narrative review of 30 studies published between 2004 and 2026 shows a rapidly evolving landscape in which generative AI, automated grading, adaptive learning, and virtual assessment environments are reshaping pharmacy-education assessment. AI tools have reached technical maturity sufficient to support meaningful roles, yet the path from capability to validated, equitable, and ethically sound deployment requires further work. Methodological limitations, gaps regarding long-term effectiveness and bias, the absence of accreditation guidance, and unresolved questions about transparency, privacy, and oversight all require concerted attention. The question is not whether AI will be integrated into pharmacy-education assessment, but how, thoughtfully, with validation, human oversight, and a commitment to educational quality and professional standards.

## Figures and Tables

**Table 1:** Comparative overview of AI assessment tool categories in pharmacy education.

Tool category	Specific tools	Primary functions	Current adoption	Key advantages	Primary limitations
Generative AI (ChatGPT/GPT-4)	GPT-4, ChatGPT-3.5/4	Grading, feedback, item creation, tutoring	Emerging/pilot	Versatile, accessible, rapid, 24/7	Accuracy variability, hallucinations, require expert validation
Automated grading systems	Custom NLP rule-based scorers	MCQ scoring, short-answer grading, competency tracking	Established	Efficiency, consistency, and immediate feedback	Limited to structured responses; setup cost
Adaptive learning platforms	IRT-based platforms, deep RL	Personalized assessment, remediation, and gap identification	Moderate	Personalized, data-rich, supports diverse paces	High cost, integration complexity, content burden
Virtual assessment environments	Virtual OSCE, AI proctoring	Clinical-skills assessment, integrity monitoring	Emerging/pilot	Scalability, flexibility, geographic access	Altered dynamics; privacy concerns; grading intensive

*Abbreviations:* IRT, item response theory; MCQ, multiple-choice question; NLP, natural-language processing; OSCE, objective structured clinical examination; RL, reinforcement learning.

**Table 2:** Summary of the 30 reviewed studies: design, findings, and limitations. Studies are identified by their reference number (superscript in the running text).

Ref.	AI tool/system	Study design	Sample/setting	Key findings	Limitations
**Grading Performance and Accuracy**					
4	GPT-4	Cross-sectional	Pharmacy students (Malaysia)	ICC 0.62–0.93 with human raters; optimized prompts improved consistency	Single institution; non–North American
15	GPT-4/LLM	Pilot	Pharmacy students (OSCE)	94%–96% grading accuracy vs human; reduced grading time	Small sample; pilot design
16	LLMs (GPT family)	Validation	Pharmacy students (critical care)	High reproducibility; accuracy 72%–87%, varying by complexity	Critical-care–specific; limited generalizability
17	GPT-3.5 vs GPT-4	Comparative	Taiwan licensure exam	GPT-4 passed (73%); GPT-3.5 failed (59%)	Single exam type; MCQ-only
18	4 AI tools	Diagnostic evaluation	Pharmacology MCQs	Accuracy 69%−88%; variable by tool and question	Pharmacology only; MCQ only
**Feedback Generation and Educational Support**					
19	AI-assisted feedback	Randomized	Pharmacy students	AI feedback more comprehensive/specific than peer; hybrid recommended	Student concerns about authenticity
20	ChatGPT	Proof-of-concept	Pharmacy students	Effective explanation; 24/7 availability valued	Potential inaccuracies; needs critical evaluation
21	AI chatbot	Development & evaluation	Students and faculty	Realistic communication practice; positive reception	Emphasizes continued human interaction
**Exam Item Creation**					
22	ChatGPT	Item generation	Medical MCQs	Rapid MCQ generation	Requires expert review; distractor quality issues
13	Generative LM	Systematic evaluation	Pharmacy exam items	Content-accurate but lacked clinical nuance	AI as starting point; refinement required
**Short-Answer Grading**					
7	Custom NLP/LLM	Validation	Pharmacology exam responses	Correlation > 0.93 with human; best for structured	Small sample; limited responses
23	Semantic similarity NLP	Comparative algorithm	E-learning short answers	Word embeddings outperformed keyword matching	Needs testing on student answers
**Reflective Writing Analysis**					
14	SVM, RF	Classification	Pharmacy reflective writing	Random-forest F-score ≈ 0.79	Below human classification
**Competency-Based Assessment Systems**					
24	Rule-based system	Early implementation	APPEs (pharmacy students)	Reduced administrative burden; improved consistency	Depended on preceptor training
**Adaptive Learning Platforms**					
9	Adaptive learning platform	Implementation	First-year pharmacy students	Positive satisfaction; self-paced learning valued	Significant faculty commitment
**Virtual OSCE Implementations**					
6	Virtual OSCE platform	Implementation & evaluation	Pharmacy students	Validity comparable to in-person; enhanced scalability	Altered dynamics; nonverbal skill limits
25	AI in pharmacy OSCEs	Qualitative	Educators & students	AI seen as augmenting, not replacing, humans	Qualitative; institutional readiness
26	Electronic OSCE scoring	Innovation report	Pharmacy faculty	Reduced transcription errors; real-time tracking	Not advanced AI; digital enhancement
**Computer-Based Testing**					
27	Computer-based testing	Experimental	Pharmacy students	Eliminating backward navigation cut time without affecting scores	Single institution; navigation-specific
5	CBT, online exams	Early implementation	Undergraduate pharmacy (Manchester)	High satisfaction; immediate feedback	Early implementation; technology evolved
28	ChatGPT	Descriptive	Pharmacy students	Active-recall practice was feasible; ease of use was reported	Single course; prompt-dependent accuracy
29	AI-supported formative	Student perspectives	Pharmacology students	Generally positive; trust/transparency concerns	Small sample; limited outcome data
**Other Reviews and Studies**					
11	AI in higher education	Meta-systematic review	Higher education broadly	Call for ethics, collaboration, and rigor	Not pharmacy-specific; broad scope
10	OSCEs (bibliometric)	Bibliometric review	Pharmacy education (20 years)	Widespread OSCE use; resource demands limit scalability	Bibliometric; not interventional
12	ChatGPT	Narrative review	Pharmacy/healthcare education	Balanced overview of promise and concerns	Narrative; digital divide
30	AI output evaluation (FLUF)	Framework development	Pharmacy education	FLUF framework for evaluating AI outputs	Framework only; needs validation
31	AI feedback trust	Commentary/analysis	Pharmacy education	Student trust in AI feedback is critical	Limited empirical data
32	ChatGPT	Descriptive	Pharmacology undergraduates	Supported learning efficiency	Limited sample; ethical considerations
33	Virtual interactive cases	Single-center experience	Pharmacy students	Enhanced clinical-reasoning practice	Single center; technical support needed
34	AI browser extension	Evaluation	Online pharmacy exams	Detected suspicious patterns	Privacy and false-positive concerns

*Abbreviations:* APPE, Advanced Pharmacy Practice Experience; CBT, computer-based testing; ICC, intraclass correlation coefficient; LLM, large language model; LM, language model; MCQ, multiple-choice question; NLP, natural-language processing; OSCE, objective structured clinical examination; RF, random forest; SVM, support vector machine.

**Table 3:** Key research gaps and recommended directions for AI assessment in pharmacy education.

Gap area	Current limitation	Recommended research direction
Longitudinal effectiveness	Most studies use short-term, pilot designs; long-term outcome data are absent	Cohort studies tracking students from AI-enhanced programs into practice
Bias and equity	Few studies examine AI performance across diverse student populations	Systematic bias testing across racial, ethnic, linguistic, and socioeconomic subgroups; diverse stakeholder involvement
Comparative effectiveness	Few studies directly compare multiple AI tools for the same task	Head-to-head trials using identical student-response datasets
Generalizability	Most studies from single institutions; many non–North American	Multi-institutional, multi-country studies; validation specific to North American PharmD contexts
Explainability/transparency	Many models operate as “black boxes”	Development and validation of explainable AI models for pharmacy assessment
Cost-effectiveness	No formal cost-effectiveness analyses	Cost-benefit analyses, including direct costs, time savings, and outcomes
Pharmacy-specific AI	Current tools are general-purpose	Pharmacy-specific models trained on curated education datasets
Faculty development	Underexplored; inadequate training is a barrier	Qualitative and mixed-methods research on faculty needs and strategies
Ethical/policy frameworks	No standardized accreditation or governance guidance	Consensus guidelines (ACPE, education societies) for validation, governance, and ethics
Student learning behaviors	Unknown how AI changes study habits	Behavioral research on learning and metacognition in AI-rich environments

**Table 4:** Future research and technology-development priorities for AI assessment in pharmacy education.

Priority area	Description	Potential impact
Pharmacy-specific AI models	LLMs/ML models trained on pharmacy education data and calibrated to ACPE competencies	Improved accuracy, fewer hallucinations, better alignment with pharmacy reasoning
Explainable AI (XAI)	Architectures providing interpretable explanations for decisions	Increased trust; improved validity evidence; more actionable feedback
Longitudinal studies	Multi-year cohorts following students into practice	Evidence on long-term learning, competency, and practice performance
Bias mitigation	Systematic testing and mitigation across populations	Equitable outcomes; protection of disadvantaged groups; compliance
Human-AI collaboration	Research on optimal division of labor between AI and humans	Efficient, fair assessment; human judgment preserved for complex competencies
Multimodal AI assessment	Integration of video, audio, and text for OSCE and skills evaluation	Enhanced assessment of interpersonal skills and professional behaviors
Adaptive assessment	Deep reinforcement learning and context-aware algorithms	Highly personalized, efficient, valid assessment across domains
Accreditation guidance	ACPE and society guidelines for validation and ethical use	Standardized framework; protection of quality and student rights
AI-literacy curriculum	Integration of AI-literacy competencies into PharmD curricula	Graduates prepared for AI-enabled practice; responsible use

*Abbreviations:* ACPE, Accreditation Council for Pharmacy Education; LLM, large language model; ML, machine learning; OSCE, objective structured clinical examination; XAI, explainable artificial intelligence.

## Data Availability

Not applicable. This manuscript is a synthesis of publicly available, peer-reviewed literature and institutional web sources; no primary data were generated.

## Abbreviations

ACPE: Accreditation Council for Pharmacy Education
AI: Artificial intelligence
APPE: Advanced Pharmacy Practice Experience
CBT: Computer-based testing
FERPA: Family Educational Rights and Privacy Act
FLUF: Flawed, Limited, Uninformative, or Fabricated
GDPR: General Data Protection Regulation
GPT: Generative pre-trained transformer
ICC: Intraclass correlation coefficient
IRT: Item response theory
LLM: Large language model
MCQ: Multiple-choice question
ML: Machine learning
NLP: Natural language processing
OSCE: Objective structured clinical examination
PharmD: Doctor of Pharmacy
RF: Random forest
RL: Reinforcement learning
SVM: Support vector machine
XAI: Explainable artificial intelligence.
